# Nicotinamide Riboside Improves Cardiac Function and Prolongs Survival After Disruption of the Cardiomyocyte Clock

**DOI:** 10.3389/fmmed.2022.887733

**Published:** 2022-05-09

**Authors:** Pieterjan Dierickx, Bryce J. Carpenter, Isaac Celwyn, Daniel P. Kelly, Joseph A. Baur, Mitchell A. Lazar

**Affiliations:** ^1^ Institute for Diabetes, Obesity, and Metabolism, Perelman School of Medicine at the University of Pennsylvania, Philadelphia, PA, United States; ^2^ Division of Endocrinology, Diabetes, and Metabolism, Department of Medicine, Perelman School of Medicine at the University of Pennsylvania, Philadelphia, PA, United States; ^3^ Cardiovascular Institute and Department of Medicine, Perelman School of Medicine at the University of Pennsylvania, Philadelphia, PA, United States; ^4^ Department of Physiology, Perelman School of Medicine at the University of Pennsylvania, Philadelphia, PA, United States

**Keywords:** NAD^+^, cardiac metabolism, circadian clock, heart failure, nuclear hormone receptors

## Abstract

The REV-ERB nuclear receptors are key components of the circadian clock. Loss of REV-ERBs in the mouse heart causes dilated cardiomyopathy and premature lethality. This is associated with a marked reduction in NAD^+^ production, but whether this plays a role in the pathophysiology of this heart failure model is not known. Here, we show that supplementation with the NAD^+^ precursor NR as a dietary supplement improves heart function and extends the lifespan of female mice lacking cardiac REV-ERBs. Thus, boosting NAD^+^ levels can improve cardiac function in a setting of heart failure caused by disruption of circadian clock factors, providing new insights into the links between the circadian clock, energy metabolism, and cardiac function.

## Introduction

The heart is a highly metabolically active organ. Many of its energy demanding pathways are under control of the circadian clock and therefore oscillate in a diurnal manner (Dierickx et al., 2017; Rana et al., 2020). The nuclear receptors REV-ERBα/β (REV-ERBs) are key regulators of the output of the circadian clock (Preitner et al., 2002; Ueda et al., 2002; Bugge et al., 2012; Cho et al., 2012). In the heart, they are essential to sustain cardiac function by driving rhythmic metabolic gene expression programs (Dierickx et al., 2022; Song et al., 2022). This rhythmicity is important, as its disruption (e.g., *via* shiftwork) is associated with adverse cardiometabolic effects (Dierickx et al., 2017).

Nicotinamide phosphoribosyltransferase (NAMPT) is the rate-limiting enzyme for NAD^+^ generation *via* the salvage pathway and is expressed in a circadian manner (Nakahata et al., 2009; Ramsey et al., 2009). Decreased cardiac levels of NAMPT as well as NAD^+^ are observed in heart failure patients (Horton et al., 2016; Lee et al., 2016; Diguet et al., 2018; Walker and Tian, 2018). *Nampt* is activated by the core clock factors BMAL1 and CLOCK (Nakahata et al., 2009; Ramsey et al., 2009), but we have recently discovered an additional role for the repressive arm of the clock pathway (Dierickx et al., 2022). The hearts of mice lacking REV-ERBs in cardiomyocytes (CM-RevDKO) constitutively express high levels of the transcriptional repressor E4BP4, which is normally expressed in a diurnal manner due to its repression by REV-ERBs (Dierickx et al., 2022). The chronic elevation of E4BP4 reduced the levels of a number of metabolic genes, such as *Nampt,* leading to decreased cardiac NAD^+^ levels in CM-RevDKO mice. This metabolic imbalance contributes to dilated cardiomyopathy and premature lethality (Dierickx et al., 2022). However, it remained unclear whether reduced NAD^+^ levels were a cause or consequence of the cardiac phenotype observed. NAD^+^ boosting strategies in cardiovascular disease have had variable results both in mice and humans (Diguet et al., 2018; Vignier et al., 2018; Walker and Tian, 2018; Abdellatif et al., 2021; Lauritzen et al., 2021; Tong et al., 2021).

Here we show that oral administration of the NAD^+^ precursor nicotinamide riboside (NR) blunts the development of cardiac dysfunction and extends lifespan in female CM-RevDKO mice. Therefore, our results reveal that low NAD^+^ levels contribute to disease progression and that NAD^+^ boosting agents alleviate the severity of the phenotype.

## Materials and Methods

### Animals

Female cardiomyocyte (CM)-specific *Rev-erbα/β* double KO (CM-RevDKO, αMHC-Cre^+^) versus Rev-erbα^ff^/β^ff^ (control, αMHC-Cre^-^) mice (Dierickx et al., 2019; 2022) were used for all experiments. They were housed under 12 h light/12 h dark conditions and fed a standard chow diet (Rodent Diet 5010, LabDiet) *ad libitum* with free access to water. NR (Elysium Health, NY, United States) was added to the drinking water (3 g/L) in light-protected bottles that were changed twice per week, starting at the age of 2 months. Whole body composition was determined by quantitative magnetic resonance using an EchoMRI body composition analyzer. All animal care and use procedures followed the guidelines of the Institutional Animal Care and Use Committee of the University of Pennsylvania.

### Quantitative RT–PCR

Total RNA was extracted from heart tissues (TRIZOL) using RNAeasy (Qiagen) according to the manufacturer’s instructions and treated with DNase (Qiagen) before reverse transcription. cDNA was generated using High-Capacity cDNA Reverse Transcription Kit (Applied Biosystems). Quantitative PCR reactions were performed using PowerSYBR Green PCR Master Mix (Applied Biosystems) with specific primers on a QuantStudio 6 Flex instrument (Applied Biosystems). mRNA expression was normalized to the housekeeping gene *Ppib* for all samples. Primer sequences for qRT–PCR: *Ppib*-fw, 5′-GCA​AGT​TCC​ATC​GTG​TCA​TCA​AG-3′; *Ppib*-rev, 5′-CCA​TAG​ATG​CTC​TTT​CCT​CCT​G-3′; *Nampt* -fw,5′-GGT​CAT​CTC​CCG​ATT​GAA​GT-3′; *Nampt*-rev, 5′-TCA​ATC​CAA​TTG​GTA​AGC​CA-3′*; Nmrk2*-fw, 5′-CAT​CTC​AGG​ACC​AGT​CAC​CT-3′; *Nmrk2-*rev, 5′-CTG​TTG​GTC​AGG​GTG​GTC​TT-3′; *E4bp4*-fw, 5′-ACG​GAC​CAG​GGA​GCA​GAA​C-3′; *E4bp4*-rev, 5′-GGA​CTT​CAG​CCT​CTC​ATC​CAT​C-3′; *Echs1*-fw, 5′-TTG​TGA​ACT​TGC​CAT​GAT​GTG​T-3′; *Echs1*-rev, 5′-TGC​TCG​GGT​GAG​TCT​CTG​AG-3′; *Dgat2*-fw, 5′-GCG​CTA​CTT​CCG​AGA​CTA​CTT-3′; *Dgat2*-rev, 5′-GGG​CCT​TAT​GCC​AGG​AAA​CT-3′; *Cpt1a*-fw, 5′-CTC​CGC​CTG​AGC​CAT​GAA​G-3′; *Cpt1a*-rev, 5′- CAC​CAG​TGA​TGA​TGC​CAT​TCT-3′; *Pgc1*-a-fw, 5′ TGT​TCC​CGA​TCA​CCA​TAT​TCC-3′; *Pgc1*-a-rev 5′-TCC​CGC​TTC​TCG​TGC​TCT​TT-3′. mRNA expression was assessed at ZT10 (5pm), except for full diurnal experiments.

### NAD^+^ Measurements

Hearts and livers were harvested at ZT10 (5pm) and snap frozen in liquid nitrogen. Hearts were powdered and subsequently lysed in 0.6 M perchloric acid. Cardiac NAD^+^ levels were measured by a cycling enzymatic assay as previously described (Dierickx et al., 2022).

### Echocardiography

Ultrasound examination of the left ventricle was performed by the Mouse Cardiovascular Phenotyping Core at the University of Pennsylvania (Cardiovascular Institute) using a Fujifilm VisualSonics Ultrasound System (VisualSonics Inc., Toronto, ON, Canada). Mice were anesthetized with an I.P. injection of 0.005 ml/g of 2% Avertin (2,2,2-Tribromoethanol, Sigma-Aldrich, St. Louis, MO, United States). Two-dimensional long-axis and short-axis M-Mode images were obtained. M Mode Images were analyzed for LV structure and function related parameters using Vevo Lab software (Visual Sonics Inc., Toronto, ON, Canada).

### Statistics

Statistical analyses were performed using Prism9 (GraphPad Software). All data are reported as mean ± SEM. Two-way ANOVA tests were used for circadian qRT-PCR data and two-sided t-tests when comparing different conditions for NAD^+^ and echocardiography data. Survival data were analyzed by using a Kaplan–Meier survival analysis with a log-rank (Mantel-Cox) test.

## Results

**FIGURE 1:**
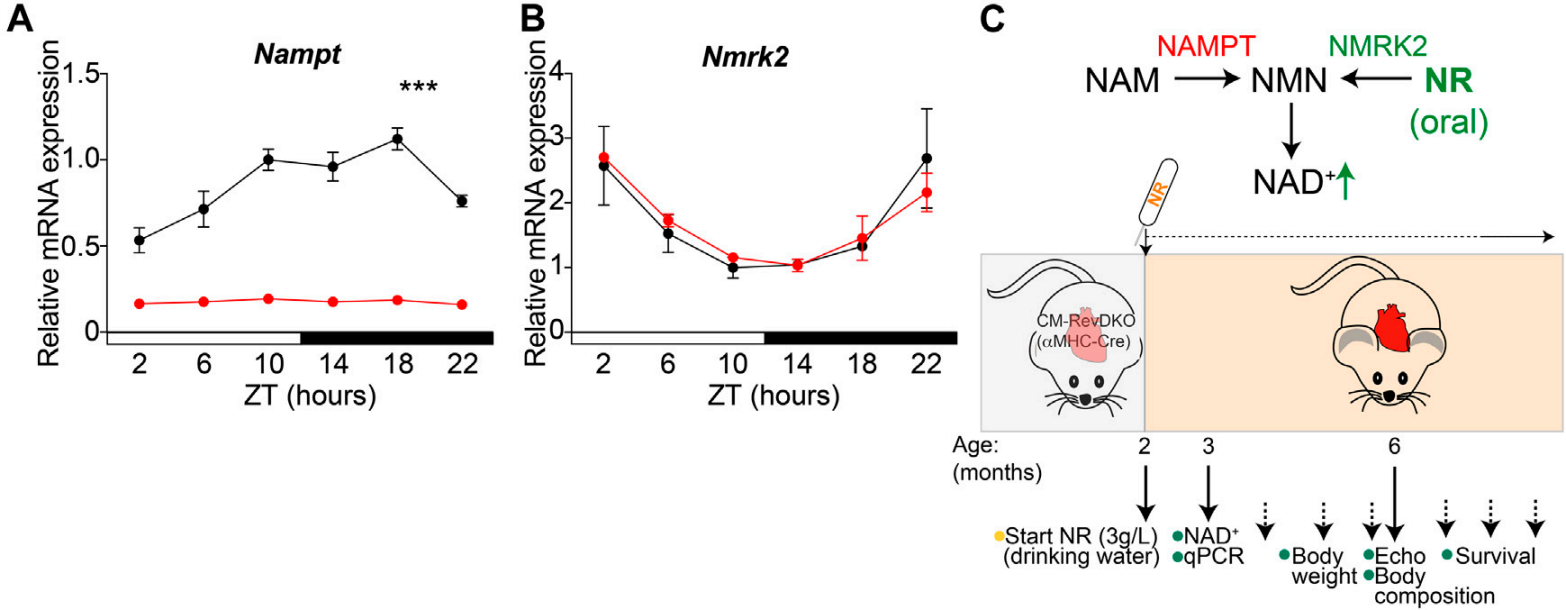
NR supplementation strategy to bypass low cardiac *Nampt* levels. **(A)** Relative *Nampt* and **(B)** *Nmrk2* mRNA expression in hearts from 2-month-old female αMHC-Cre^+^ *Rev-erb*α/β double floxed (CM-RevDKO) vs littermate controls (αMHC-Cre^-^ *Rev-erb*α/β double floxed) (*n* = 3 hearts/genotype/timepoint; ***p < 0.001 by two-way ANOVA). **(C)** Schematic representation of NAD^+^ boosting strategy in CM-RevDKO mice with NR.

To determine whether reduced NAD^+^ levels are in part causal to observed cardiac dysfunction in CM-RevDKO animals (Dierickx et al., 2022), we investigated the potential beneficial effect of supplementation with the NAD^+^ precursor (nicotinamide riboside, NR). As cardiac enlargement and lethality were more pronounced in female than in male mice (Dierickx et al., 2022), we focused on females and confirmed drastically reduced and non-rhythmic cardiac *Nampt* mRNA levels across the circadian cycle in CM-RevDKOs (Figure 1A). In contrast, *Nmrk2*, another important NAD^+^ biosynthetic enzyme in the heart displayed unchanged expression levels and remained circadian after CM-RevDKO (Figure 1B). Since NRK2 (encoded by *Nmrk2*) catalyzes the first step of NR to NAD^+^, bypassing the need for NAMPT, we added its substrate, NR, to the drinking water (3 g/L) starting at the age of 2 months when mice did not yet show major structural or functional cardiac defects (Dierickx et al., 2022) (Figure 1C).

**FIGURE 2:**
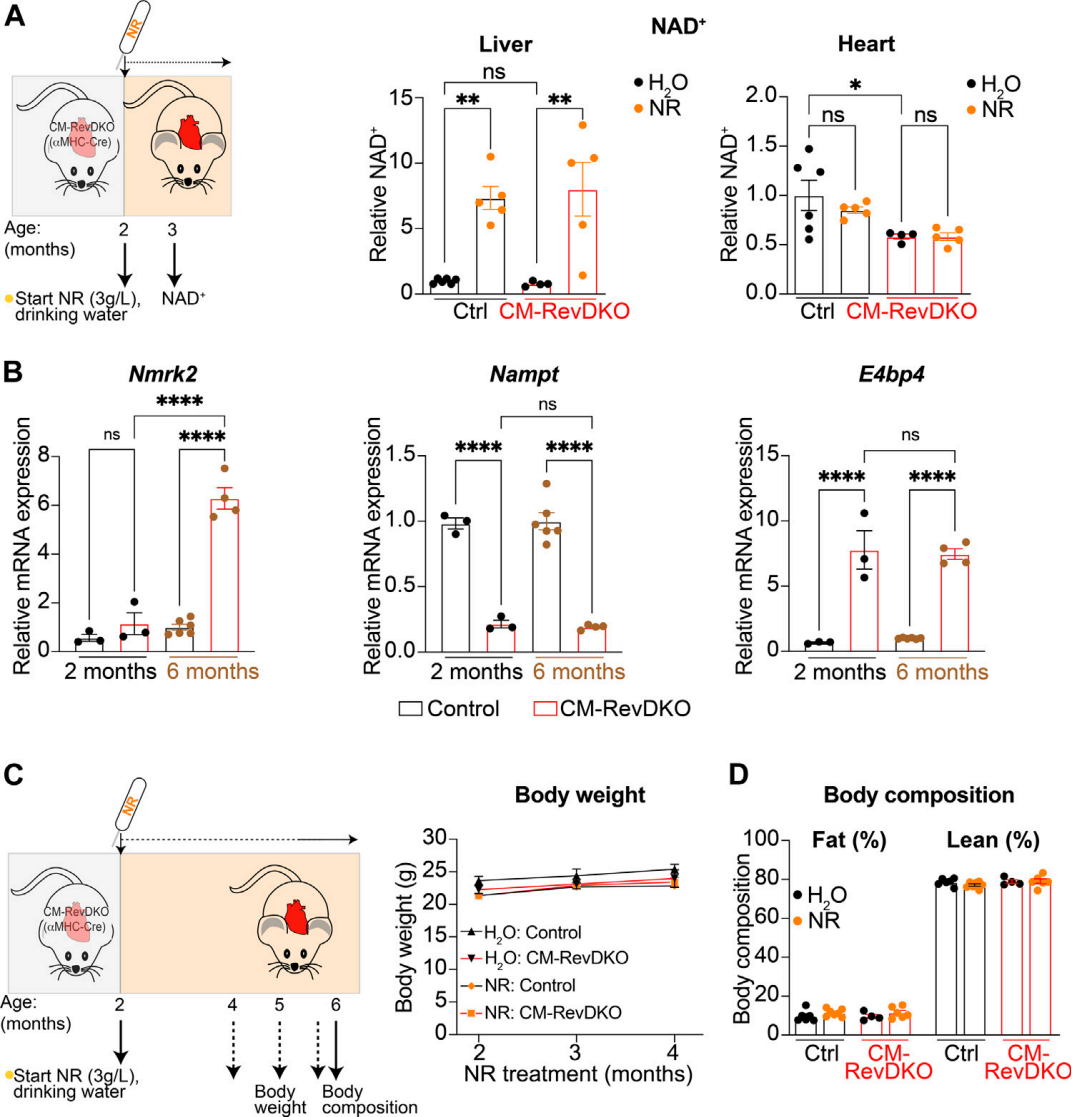
NAD^+^ levels after 1-month NR treatment and its long-term effect on body weight and composition. **(A)** Schematic representing NR treatment and NAD^+^ concentration in livers and hearts of control versus CM-RevDKO female mice treated with NR versus H_2_O (control) for 1 month (*n* = 4–6/genotype/treatment). **(B)** mRNA expression in hearts from 2-month- and 6-month-old female CM-RevDKO vs controls (*n* = 3–6 hearts/genotype; ns, not significant; *p < 0.05, **p < 0.01, ****p < 0.0001 by one-way ANOVA, followed by a Šidák’s multiple comparisons test). **(C)** Schematic representing NR treatment and body weight and **(D)** body composition of female control vs CM-RevDKO mice treated with NR for 4 months, starting at an age of 2 months (*n* = 4–7/genotype/treatment).

We measured NAD^+^ levels in liver and heart after 1 month of NR treatment and while hepatic NAD^+^ levels increased drastically, validating our supplementation strategy, cardiac NAD^+^ levels did not change at this time point (Figure 2A). This is in line with a previous report (Lauritzen et al., 2021), and is likely due to high cardiac metabolic turnover (Trammell et al., 2016). In addition, *Nmrk2* levels are known to be increased in heart failure (Diguet et al., 2018) and young (2-month-old) CM-RevDKO mice do not show signs of severe heart failure yet (Dierickx et al., 2022). In contrast, we find *Nmrk2* levels of 6-month-old DKO mice to be significantly higher compared to young mice, while *Nampt* and *E4bp4* levels are the same (Figure 2B). This suggests that as they age, CM-RevDKO mice potentially benefit more from NR treatment due to higher *Nmrk2* levels. Body weight and composition of control and CM-RevDKO mice were not affected (Figures 2C,D), suggesting no detrimental systemic effect of the NR treatment itself.

**FIGURE 3:**
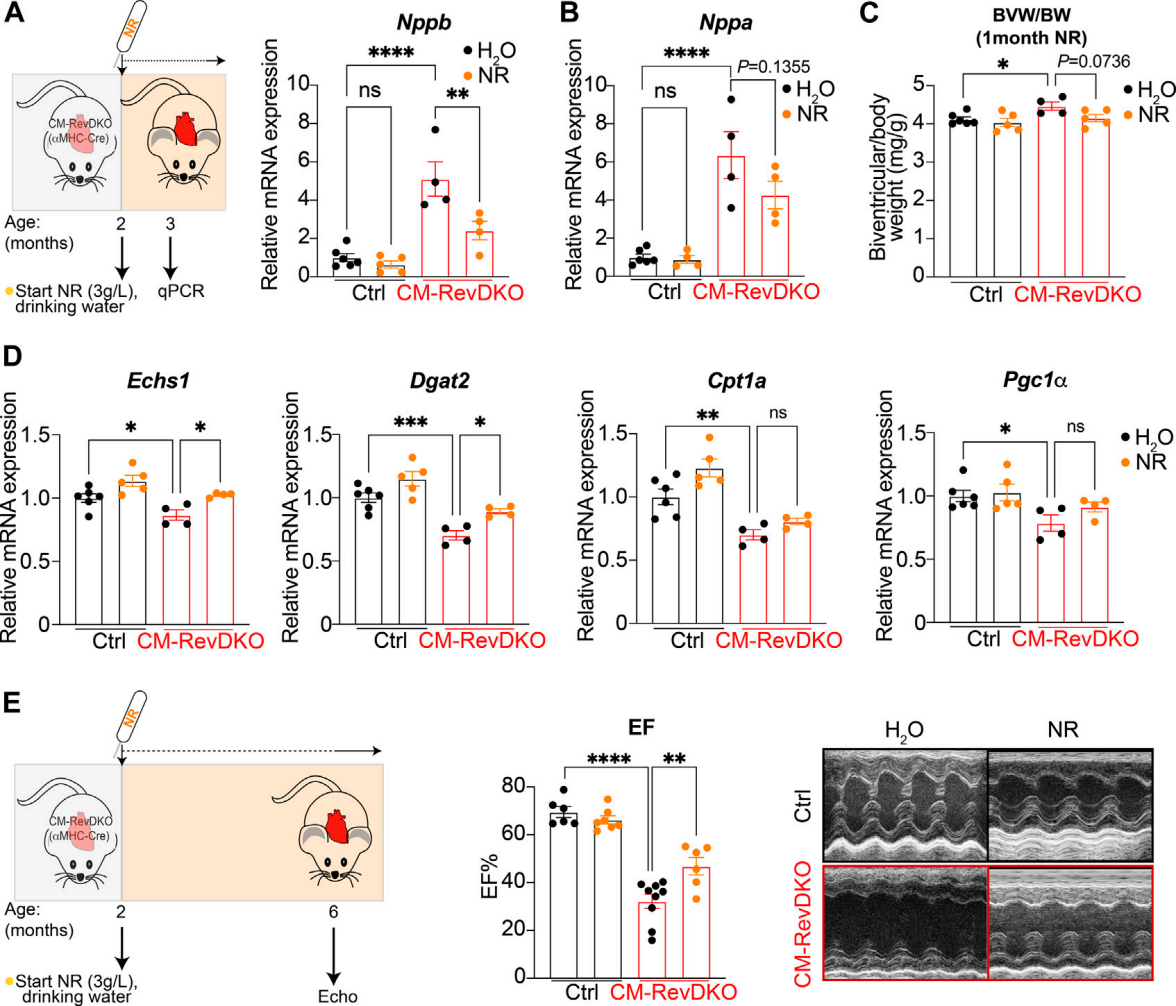
NR supplementation alleviates cardiac stress and improves heart function in female CM-RevDKO mice. **(A)** Schematic representing NR treatment and relative *Nppb* and **(B)** *Nppa* mRNA expression in hearts from 3-month-old female CM-RevDKO vs controls treated with NR for 1 month (*n* = 4–6 hearts/genotype/treatment). **(C)** Biventricular to body weight (BVW/BW) ratio from mice in **(A**,**B)**. **(D)** mRNA expression in hearts from 3-month-old female CM-RevDKO vs controls treated with NR for 1 month (*n* = 4–6 hearts/genotype/treatment; ns, not significant; *p < 0.05; **p < 0.01; ***p < 0.001; ****p < 0.0001 by one-way ANOVA, followed by a Šidák’s multiple comparisons test). **(E)** Schematic representing NR treatment and cardiac function: EF: Left ventricular ejection (%) fraction data determined by echocardiagraphy for 6–7-month-old female mice and (right) representative short axis M-mode images from echocardiography on mice from the (left). (*n* = 6–9/condition; 2 CM-RevDKO animals on H_2_O (control) died before they could be echoed; ns, not significant; **p < 0.01; ****p < 0.0001 by 2-sided Student’s t test.

While no overt phenotype was observed at the age of 3 months in CM-RevDKO mice, cardiac stress markers (*Nppa* and *Nppb* encoding ANP and BNP respectively) are upregulated at this time point, indicating that disease development had initiated. Upon NR treatment for 1 month (started at the age of 2 months), levels of *Nppb*, but not *Nppa,* were significantly lower in CM-RevDKO animals, indicating discernable alleviation of cardiac stress (Figures 3A,B). Consistent with this, we noted that the mild enlargement of CM-RevDKO hearts was reduced upon NR treatment, albeit these results did not reach significance (Figure 3C, *p* = 0.074). Some metabolic genes downregulated in CM-RevDKO mice, such as those encoding mitochondrial proteins (*Echs1* and *Dgat2*), increased significantly upon NR supplementation in DKO hearts while others did not (*Cpt1a* and *Pgc1α*, Figure 3D). We thus focused on a later time point (∼6–7 months) at which heart function is significantly affected in CM-RevDKO mice (Dierickx et al., 2022). Echocardiography revealed a marked improvement of left ventricular ejection fraction upon 4 months of NR treatment in these animals (Figure 3E).

**FIGURE 4:**
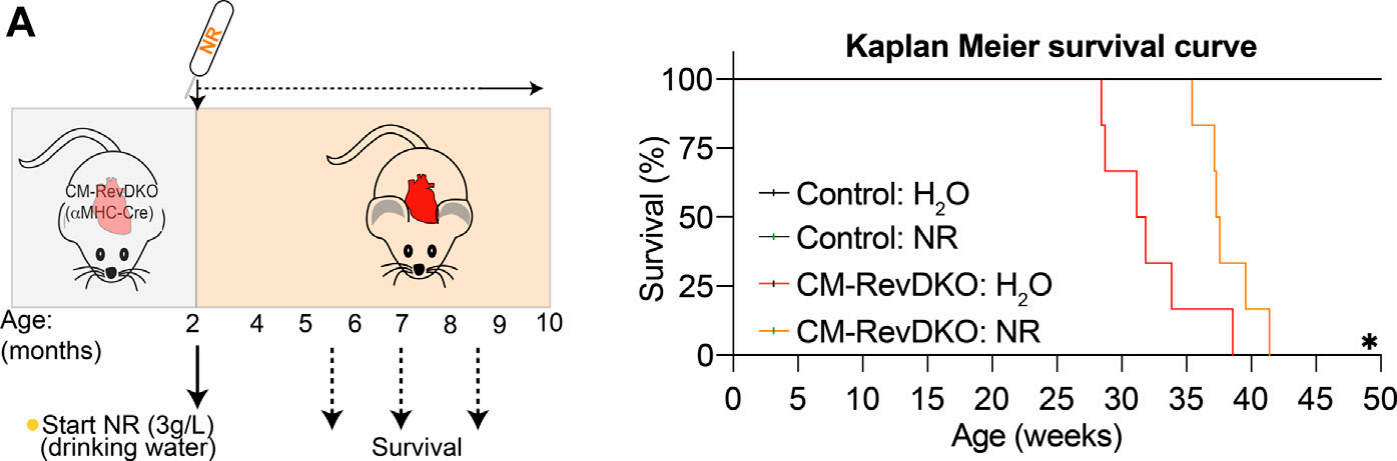
NR supplementation extends median lifespan of female CM-RevDKO mice. **(A)** Schematic representing NR treatment and Kaplan-Meier survival curves for control and CM-RevDKO female mice treated without (H_2_O) or with NR (*n* = 6–7/condition). *p < 0.05 by log-rank (Mantel-Cox) test.

We next aged the mice to investigate survival rate. NR treatment lengthened median survival by nearly 6 weeks (Figure 4A). The lack of complete rescue of the cardiomyopathy by NR is consistent with additional defects in fatty acid oxidation and mitochondrial function in CM-RevDKO mice (Dierickx et al., 2022). Thus, while NR-treated female mice still die prematurely, cardiomyopathy and lethality due to loss of REV-ERBs is significantly ameliorated by supplementation with NAD^+^ precursors.

## Discussion

Abnormal circadian clocks have been associated with cardiovascular disease in humans. Cardiovascular diseases have themselves been associated with impaired NAMPT/NAD^+^ metabolism, and some but not all studies have demonstrated a benefit of restoring NAD^+^ levels in heart failure. None of these studies have evaluated heart failure due to circadian clock dysfunction. Here, using a recently described mouse model of dilated cardiomyopathy with impaired NAD^+^ metabolism due to a dysfunctional cardiac circadian clock (lack of REV-ERB nuclear receptors), we show that treatment with the NAD^+^ precursor, NR, improves cardiac function and mortality. To our knowledge, this is the first study to show that NR can improve heart function specifically in female mice. Future studies will address whether NAD^+^ supplementation is also effective in male CM-RevDKO mice.

We have previously shown that E4BP4-mediated *Nampt* repression is a common mechanism in cardiomyocyte-specific *Rev-erbα/β* DKO as well as *Bmal1* KO (CBK) hearts. Therefore, it would be interesting to test whether NAD^+^-boosting strategies in CBK animals will have similar cardioprotective effects.

In addition, more precise dosing of NAD^+^-boosting agents should be explored, since high levels of NR are reported to cause irregular cardiac mitochondrial morphology as evidenced by rounder mitochondria with fewer inter-mitochondrial junctions. Since mitochondrial structure and function are coupled, it could be that high levels of NR potentially lead to impaired cardiac function (Lauritzen et al., 2021). After 1 month of NR treatment intracellular cardiac NAD^+^ levels were not increased in young CM-RevDKO female mice. This is in line with the notion that increased NAD^+^ biosynthetic flux does not always lead to net increased total NAD^+^ levels since these are a result of synthesis and consumption (Gardell and Coen, 2022). In addition, increasing *Nmrk2* levels during disease progression (Figure 2B) might further influence dosing strategies. Nevertheless, our results show that boosting NAD^+^ levels can improve cardiac function in a setting of heart failure caused by disruption of circadian clock factors, which provides a new perspective on the links between the circadian clock, energy metabolism, and cardiac function.

## Data Availability

The original contributions presented in the study are included in the article. Further inquiries can be directed to the corresponding authors.
